# The analysis of fertility quality of life and the influencing factors of patients with repeated implantation failure

**DOI:** 10.1186/s12955-021-01666-3

**Published:** 2021-01-25

**Authors:** Ying Ni, Chenye Tong, Limin Huang, Wenjie Zhou, Aijun Zhang

**Affiliations:** 1 Nursing Department of Ruijin Hospital Affiliated to Shanghai Jiaotong University School of Medicine, 197 Ruijin 2nd Road, Shanghai, 200025 China; 2 Reproductive Medical Center of Ruijin Hospital Affiliated to Shanghai Jiaotong University School of Medicine, 197 Ruijin 2nd Road, Shanghai, 200025 China

**Keywords:** Repeated implantation failure, Fertility quality of life, Social support, Anxiety, Depression, Influencing factors

## Abstract

**Background:**

The objective of this study was to investigate the current status of fertility quality of life (QoL) and explore the influencing factors for infertile women with repeated implantation failure (RIF).

**Methods:**

The sample consisted of 137 infertile women with RIF who were under treatment from January 2019 to December 2019 in the Reproductive Medical Center of Ruijin Hospital, affiliated with Shanghai Jiaotong University School of Medicine in China. A general information questionnaire, FertiQoL scale, perceived social support scale (PSSS), self-rating anxiety scale (SAS), and self-rating depression scale (SDS) were used to analyse the fertility QoL and related factors of RIF patients.

**Results:**

The total fertility QoL score of RIF patients was 60.44 ± 11.60. The results of multivariate regression analysis showed that residence, financial difficulties, male infertility, BMI index, depression, and family social support were the main factors that influenced the fertility QoL of RIF patients (adjusted R^2^ = 0.762).

**Conclusion:**

Based on the results of this study, RIF patients’ psychological status must be addressed. Corresponding interventions such as building a sound family and social support system, creating a good medical environment and offering diverse health education should be provided to improve the fertility QoL of RIF patients.

## Background

With changes in the living environment, the accelerating pace of life and delays in marriage and childbearing, the incidence of infertility is rising year by year. According to statistics from the World Health Organization (WHO), approximately 8–12% [1] of couples worldwide have experienced infertility. Since the approaches of controlled ovarian stimulation (COS) and the conditions of the embryonic laboratory have improved, in vitro fertilization-embryo transfer (IVF-ET), as the main assisted reproductive therapy for infertile patients, brings hope to many infertile patients. However, despite high-quality embryos, many patients remain whose embryos cannot be implanted normally for various reasons. Repeated implantation failure (RIF) refers to failure to conceive after three or more in vitro fertilization (IVF) cycles, intracytoplasmic sperm injection (ICSI) embryo transfer cycles, or frozen-thawed embryo transfer cycles or four or more high-quality embryo transplantation cycles [2]. The incidence of RIF reaches 5%–10% during IVF/ICSI-assisted pregnancy treatment [3].

Infertility is one of the greatest stressors in life and results in psychological distress [4]. It can lead to negative psychological consequences and diminished quality of life and well-being [5]. Studies have shown that a poor psychological state can reduce the pregnancy rate of IVF-ET and can have adverse effects on pregnancy outcome [6]. The QoL of women with infertility is obviously lower than that of other women of childbearing age [7]. The decrease in patients' QoL affects patient treatment compliance, thereby affecting the pregnancy rate [8]. Coughlan et al. [9] compared psychological stress among women with and without RIF and found that it was significantly higher among women with RIF. As repeated failure of infertility, RIF causes a heavy financial burden and mental stress on patients and families and affects their quality of life (QoL) [10].

Recent research shows that the influencing factors of the QoL of women with infertility mainly include age, gender, educational level, duration of infertility, fertility expectation, psychological status, marital relationship, and history of assisted pregnancy. According to Hassanin [11] and Karabulut et al. [12], women with secondary infertility and with higher education levels have higher fertility QoL, and the duration of infertility and desire for psychological support are negatively correlated with QoL. According to Chachamovich [13], low educational level, strong desire to have children, poor marriage relationship, history of assisted pregnancy and long duration of infertility reduce the QoL of women with infertility. Rashidi [14] showed that the duration of infertility or the attribution of infertility had no effect on health-related QoL in infertile patients.

QoL is also one of the main indexes to assess the effects of medical and nursing work. It has become one of the standards of nursing to include a QoL assessment in the clinical treatment of infertility problems [15]. However, to the best of our knowledge, there is little research focused on the QoL specifically in case of RIF patients. The purpose of this study was to investigate the current status of the fertility QoL of RIF patients and to discuss the influencing factors on their fertility QoL, thereby providing a scientific basis for effective clinical interventional measures and helping to improve the fertility QoL of RIF patients.

## Methods

### Ethics statement

The study protocol was in accordance with the ethical standards and was approved by the Ethics Committee of Shanghai Ruijin Hospital. Written informed consent was obtained from each participant. Information collected from all participants was kept confidential and anonymous.

### Data and study design

This study was conducted among outpatient women diagnosed with infertility with RIF from January to December 2019. All participants were recruited at the Center of Reproductive Clinic, Ruijin Hospital, Shanghai Jiaotong University School of Medicine in China. The inclusion criteria were as follows: ① women outpatients diagnosed with RIF, which is defined by Coughlan [2] and others as ≥ 3 cycles, ≥ 4 high-quality embryos, age < 40 years old; ② women who signed informed consent after completely comprehending the contents of the study; ③ women who had basic ability to read, communicate, and complete the questionnaire independently. The exclusion criteria were as follows: ① women diagnosed with previous or current mental disorders, cognitive impairment, or inability to understand the content of the questionnaire; ② women diagnosed with severe chronic diseases; ③ women who had recently undergone major domestic affairs.

After written informed consent was obtained for this study, a self-reported questionnaire was distributed to each eligible participant, and clinical data were collected from their medical records. Questionnaires with any missing data were excluded from statistical analyses. All questionnaires were required to be completed independently by the patients. The questionnaires were collected, and their completeness was checked on the spot. The investigation process was conducted anonymously. The questionnaire elimination criteria were as follows: ① the same answer for each question or ② withdrawal from this research for various reasons. A total of 150 questionnaires were sent out and all of them were received, for a response rate of 100%. Thirteen questionnaires were excluded due to missing answers, the same answers for each question, or deviation from the inclusion criteria. In total, 137 complete responses were received in the present study. The effective rate of the questionnaires was 91.33%.

## Measures

### Demographic characteristics

The demographic characteristics were collected with a general information questionnaire designed by our panel, which included age, height, weight, residence, occupation, education level, monthly household income, attribution of infertility, types of infertility, years of infertility, and number of treatment cycles.

### Measurement of fertility QoL

The simplified Chinese version of the FertiQoL Scale was used to measure fertility QoL in women with infertility with RIF in this study. The FertiQoL Scale was designed by experts from the European Society of Human Reproduction and Embryology and the American Society of Reproductive Medicine [16]. It is used to measure the fertility QoL of infertile patients during the treatment period. The scale has been translated into more than 20 languages. It is widely used among infertile patients in different countries and regions of the world and has good reliability, validity, and sensitivity [17, 18]. The scale is divided into two parts: a core module and an optional treatment module. There are 36 items in total, including 2 independent items of subjective general health status and subjective overall QoL and 24 core items including affective responses, physical and mental relationships, marital relationships, and social relationships. The 10 optional treatment items include treatment tolerance and treatment environment. The FertiQoL Scale is scored with 5 grades; each item is scored from 0 to 4. Of these, 7 items are reverse scored. The original score is calculated by adding the scores of each item, which is standardized to a 100-point system. The standard score is calculated as follows: original total score * 25/the number of items[16]. In the present study, the Cronbach’s alpha coefficient of the FertiQoL Scale was 0.921.

### Measurement of social support

The Chinese version of the Perceived Social Support Scale (PSSS), designed by Zimet et al. [19] in 1988, was used to assess social support. The PSSS is a social support scale that emphasizes individual self-understanding and feelings. It measures the degree of social support perceived by the individual, such as from family, friends and others. The total score reflects the overall level of social support perceived by the individual. The scale consists of 12 self-rating items, including 3 subscales: family support, friend support, and other support (teachers, classmates, relatives). Each item is scored from 1 to 7, with "Strongly disagree" as 1 point and "Strongly agree" as 7 points. The total score is 12–84. The higher the score of each dimension and the overall level, the higher the level of social support: 12–36 points indicate low support. 37–60 points indicate intermediate support, and 61–84 points indicate high support. The scale is widely used in various fields and has been proven to have good reliability and validity. The Cronbach’s alpha coefficient of the PSSS was 0.941 in this study.

### Measurement of anxiety

The Self-rating Anxiety Scale (SAS), developed by Zung [20] in 1971, is used to measure the degree of anxiety in adults. In this study, the infertility-related anxiety of participants was measured with the Chinese version of the SAS. The SAS has 20 items, each of which has a 4-level score: "1" = no or seldom; "2" = sometimes; "3" = most of the time; and "4" = most or all of the time. Five items are scored inversely. The score of each item is added for the initial score and then multiplied by 1.25 to obtain the standard score. Mild anxiety is indicated by a standard score of 50–59 points, moderate anxiety is indicated by a standard score of 60–69 points, and severe anxiety is indicated by more than 70 points. The Cronbach’s alpha coefficient of the SAS was 0.897 in this study.

### Measurement of depression

The Self-rating Depression Scale (SDS), developed by Zung [21] in 1965, is used to measure the severity of depression in adults. We used the Chinese version of the SDS to measure the infertility-related depression of participants in this study. The SDS has 20 items, each of which is assessed in 4 grades based on the following criteria: "1" = no or seldom; "2" = sometimes; "3" = most of the time; and "4" = most or all of the time. Ten items are scored inversely. At the end of the assessment, the scores of the 20 items are added for the initial score and then multiplied by 1.25 to obtain the standard score. Mild depression is indicated by a standard score of 50–59 points, moderate depression is indicated by a standard score of 60–69 points, and severe depression is indicated by more than 70 points. The Cronbach’s alpha coefficient of the SDS was 0.885 in this study.

### Statistical analysis

In this study, statistical analysis was performed with SPSS 23.0 software. The measurement data are presented as the mean ± standard deviation, and the enumeration data are expressed as the frequency and constituent ratio (%). The difference in the demographic and disease characteristic variables of the FertiQol was tested by an independent-sample t-test and single-factor variance analysis. Fertility QoL, social support, anxiety, and depression were analysed by Pearson correlation analysis. The influencing factors of fertility QoL were analysed by multiple linear regression analysis. P < 0.05 was considered statistically significant.

## Results

### Description of the participants and fertility QoL

The ages of the 137 patients ranged from 26 to 39 years old, with an average age of 32.80 ± 3.63 years. BMI (body mass index) was 17.36–33.95 kg/m^2^, with an average BMI of 21.95 ± 3.08 kg/m^2^. The average infertility period was 5.39 ± 2.87 years, ranging from 1 to 14 years. The number of treatment cycles was 3–6, and the average number of treatment cycles was 2.01 ± 0.84.

The total fertility QoL score of RIF patients was 60.44 ± 11.60 points. The score of the core module was 59.80 ± 13.90. The score of the treatment module was 61.99 ± 10.65. The dimensions were arranged according to the score from high to low: treatment environment, social relationship, marriage relationship, emotional reaction, treatment tolerance, and physical and mental relationships. Details are presented in Table 1.

**Table 1:** Total Fertility QoL Score and Each Dimension Score of RIF Patients (n = 137)

Item	Entries	Score (x ± s)
Therapeutic environment	6	66.88 ± 11.72
Social relations	6	64.78 ± 18.13
Marital relations	6	63.96 ± 12.53
Emotional response	6	56.17 ± 17.05
Treatment tolerability	4	54.65 ± 15.51
Relationship between body and mind	6	54.29 ± 17.96
Total Score of Core Module	24	59.80 ± 13.90
Total Score of Treatment Module	10	61.99 ± 10.65
Total Score of Fertility QoL	34	60.44 ± 11.60

### Social support, anxiety and depression

The social support score of RIF patients in this study was 60.92 ± 12.02 points. The scores for family support, friend support and other support were 21.31 ± 5.01, 19.78 ± 4.60 and 19.83 ± 4.03, respectively. The anxiety score was 54.84 ± 9.79, and the depression score was 58.22 ± 9.99. Ninety-five out of 137 RIF patients had an anxiety state that was 69.34% of the incidence rate and 57.13 ± 9.08% of the fertility QoL score. There were 114 patients with depression. The incidence rate was 83.21%, and the fertility QoL score was 58.18 ± 8.68. Anxiety and depression status are detailed in Table 2.

**Table 2:** Anxiety and Depression Status (n = 137) and Fertility QoL Score of RIF Patients

	Total score x ± s	Number n	Incidence%	Mild %	Moderate-severe %	Fertility QoL score x ± s
Anxiety	54.84 ± 9.79	95	69.34 (95/137)	33.58 (46/137)	35.77 (49/137)	57.13 ± 9.08
Depression	58.22 ± 9.99	114	83.21 (114/137)	36.50 (50/137)	46.72 (64/137)	58.18 ± 8.68

### Univariate analysis of fertility QoL

The total scores for the fertility QoL of RIF patients differed in the following factors: BMI segment, educational level, residence, years of infertility, employment status, monthly household income, self-assessment of financial difficulties, purpose of assisted pregnancy, attribution of infertility, and number of treatment cycles (P < 0.05).

### Multivariate linear regression analysis on the influencing factors

The fertility QoL score was regarded as a dependent variable, and the variables with statistical significance in univariate analysis were included in the regression model. As shown in Table 5, the results of multivariate regression analysis showed that residence, financial difficulties, male infertility, BMI index, depression, and family social support were the main factors that influenced the fertility QoL of RIF patients (adjusted R^2^ = 0.762).

## Discussion

### Current status of fertility QoL in RIF patients

Karabulut et al. [22] used the FertiQoL scale to study the fertility QoL of women with IVF. The results showed that the average fertility QoL score of women with infertility was 66. This study used the FertiQoL scale to investigate RIF patients in our centre. The average fertility QoL score was 60.44 ± 11.60, and the average score range of each dimension was 54.29–66.88. Compared with the Karabulut study, the fertility QoL and scores of each dimension of RIF patients in this study were reduced to varying degrees. Ying L Y et al. [23] reported that the fertility QoL is significantly lower than that of women of childbearing age without infertility. Factors including the specific Chinese cultural background, traditional concepts, heavy financial burden and social public opinion lead to low QoL on infertile women. RIF patients, as part of infertile women, may suffer more economic pressure, physical pain, psychological distress, and disappointment caused by repeated failure during treatment.

In this study, among the dimensions of fertility QoL, the scores of physical-mental relationship and treatment tolerance were lower in RIF patients. The long-term, complicated treatment process, continuous drug use, surgical treatment and other invasive treatments cause physical pain, financial pressure, and psychological shock to RIF patients, which result in a sharp decline of their QoL. In addition, RIF patients are under pressure from their spouses and family members. As couples are daunted by repeated treatment failure, their emotional communication worsens; the resultant marriage crisis may cause them to fail to obtain sufficient family and social support, which damages the fertility QoL. Among the dimensions, the treatment environment ranked the highest. By optimizing the treatment environment and process, formulating a proper set of treatment and nursing measures, increasing patients' trust in medical institutions and professionals and reducing the negative emotions and psychological pressure of RIF patients, we can enhance the therapeutic effects and improve their fertility QoL.

### Analysis of influencing factors of fertility QoL

#### Residence

This study found that RIF patients in urban areas exhibit higher fertility QoL than patients in rural areas (Table 3). Patients in rural areas maintain relatively old-fashioned ideas and are more influenced by the traditional Chinese saying, "There are three forms of unfilial conduct, of which the worst is to have no descendants". In China's rural areas, children are the link to family. It is an important task for most rural families to have a child and carry on the family line. They tend to attribute infertility to women and place a greater mental burden on infertile women with RIF, whose QoL is consequently severely impaired. Moreover, rural patients with RIF must travel to urban areas far from home for examinations and treatments, which causes them hardships, such as additional transportation and accommodation costs. In contrast, despite the rapid pace of life, high pressure and irregular routine, women in urban cities are relatively more independent and well paid and generally have a later childbearing age. Furthermore, urban society is more tolerant and open to women with infertility. All of these factors may be responsible for the high fertility QoL scores in urban RIF patients.

**Table 3:** Univariate Analysis of Influencing Factors of Fertility QoL of RIF Patients (x ± s)

Variables	Group	Proportion%	Total Score of Fertility QoL		
			Score	t/F	P
Age (years)	≤ 30	42 (30.66%)	58.84 ± 10.37	1.583	0.209
	31–35	56 (40.88%)	59.76 ± 12.19		
	36–40	39 (28.47%)	63.16 ± 11.81		
BMI index	< 18.5	6 (4.38%)	41.30 ± 9.89	10.147	0.000
	18.5–23.9	108 (78.83%)	60.91 ± 11.06		
	≥ 24	23 (16.79%)	53.27 ± 11.60		
Educational level	Junior high school or below	25 (18.25%)	56.24 ± 13.25		
	Senior high school/Technical secondary school	17 (12.41%)	65.23 ± 10.96	8.913	0.000
	Junior College/Undergraduate	83 (60.58%)	59.71 ± 11.31		
	Master’s degree or above	12 (8.76%)	67.53 ± 4.29		
Residence	City	76 (55.47%)	61.19 ± 12.63	3.970	0.025
	Town	43 (31.39%)	61.66 ± 9.79		
	Rural	18 (13.14%)	54.41 ± 9.54		
Years of infertility (years)	≤ 3	36 (26.28%)	67.46 ± 8.50	13.703	0.000
	4–5	44 (32.12%)	57.15 ± 11.46		
	≥ 6	57 (41.61%)	58.55 ± 11.70		
Employment status	Rest	51 (37.23%)	56.00 ± 11.74	0.642	0.000
	On-the-job	86 (62.77%)	63.08 ± 10.73		
Time-off or not	No	59 (43.07%)	60.92 ± 13.34	8.384	0.692
	Yes	78 (56.93%)	60.09 ± 10.16		
Family monthly income	≤ 10,000	69 (50.36%)	54.24 ± 8.54	42.215	0.000
	10,001–15,000	38 (27.74%)	62.27 ± 10.86		
	> 15,000	30 (21.90%)	72.40 ± 8.10		
Self-assessment of financial difficulties	No difficulties	38 (27.74%)	71.01 ± 7.91	58.216	0.000
	Slight difficulties	70 (51.09%)	59.71 ± 9.04		
	Very difficult	29 (21.17%)	48.38 ± 8.10		
Purpose of assisted pregnancy	Cephalic foetus	124 (90.51%)	59.37 ± 11.30	0.734	0.001
	Second foetus	13 (9.49%)	70.70 ± 9.43		
Type of infertility	Primary infertility	76 (55.47%)	59.39 ± 12.37	4.100	0.226
	Secondary infertility	61 (44.53%)	61.77 ± 10.50		
Attribution of infertility	Female factors	72 (52.55%)	58.12 ± 12.69	3.251	0.046
	Male factor	20 (14.60%)	62.98 ± 8.28		
	Bilateral factors	45 (32.85%)	63.04 ± 10.38		
Number of treatment cycles	3	47 (34.31%)	63.77 ± 8.91	16.322	0.000
	4	41 (29.93%)	64.94 ± 11.55		
	≥ 5	49 (35.77%)	53.50 ± 10.80		

Note: * indicates P < 0.05

### Attribution of infertility

Infertility is often considered to be a decrease or loss of fertility. Therefore, patients with male infertility may suspect their masculinity, especially men with sexual dysfunction or azoospermia who may have strong feelings of inferiority and guilt [24]. This study found that male infertility is one of the factors that affects the QoL of infertile women with RIF (Table 5). It is likely that the negative emotions and personality changes related to male infertility lead to a lack of timely support, encouragement and comfort to RIF patients. Due to the lack of care from their husbands, the fertility QoL of women with RIF is low. As health care professionals, we should evaluate couples systematically to fully understand their emotional states. In addition, joint treatment and positive intervention for the couple are necessary to solve couples’ psychological problems and improve the state of communication in couples. The ultimate goal is to enhance the marriage satisfaction of the couple. On the one hand, men are encouraged to support and accompany their wives. On the other hand, the couple should fully understand that reproduction is the responsibility of both parties, which requires mutual understanding and support as well as joint efforts.

### Financial difficulties

Chachamovich et al. [25] reviewed the literature published in 1980–2009 on the factors that influence the QoL of patients with infertility and found that low income of women with infertility is a predictor of lower QoL. Keramat et al. [26] used the World Health Organization Quality of Life Brief (WHOQoL-BREF) and FertiQol to investigate 385 infertile couples and found that body section of the WHOQoL-BREF was significantly correlated with economic income. In the emotion/body part of the FertiQol subscale, the low-income group scored 49.82 points while the high-income group scored 56.08 points, with significant differences. In this study, monthly household income and financial difficulties were influencing factors of QoL (Table 5), which was consistent with the results of Chachamovich and other studies. The lower the family income, the lower the fertility QoL score. Moreover, there is a significant difference in the QOL scores of patients with high or low monthly family income (Table 3). In addition, failures in repeated implanting and enormous medical expenses exacerbate the financial burden, resulting in a decrease in QoL. Patients with low income must consider the basic needs of the family before the cost of assisted reproductive treatment, which affects QoL, while patients with high income can obtain more medical resources and receive better treatment. For families with financial difficulties, medical institutions can seek charity sponsorship and other means to realize patients’ dreams of assisted pregnancy, thereby improving the QoL of these patients.

### BMI Index

The body mass index (BMI) is a relative reference standard recommended by the WHO to assess the weight status of the body and is one of the important indicators for evaluating the fertility of women of childbearing age. Some scholars believe that overweight and obesity affect the quality of embryos and lead to many problems in the process of in vitro fertilization, such as high gonadotropin consumption, a decreased number of ova, a lower rate of high-quality embryos and clinical pregnancy, and an increased rate of abortion [27–29]. However, other researchers believe that overweight and obesity have no negative impact on the outcome of in vitro fertilization-embryo transfer therapy and do not affect the pregnancy outcome of in vitro fertilization [30, 31]. The results from our study revealed that RIF patients with a BMI ≥ 24 kg/m^2^ had lower QoL scores than those with a BMI in the normal range of 18.5–23.9 kg/m^2^ (Table 3). Similar to the study of Nanette Santoro, who compared 733 patients with polycystic ovary infertility and 865 patients with unexplained infertility [32], the higher the BMI of women with polycystic ovary is, the lower their fertility QoL scores are. It has been found that overweight is an important factor in anxiety and depression [33, 34], which is caused not only by external appearance characteristics but also by pathological changes. These patients exhibit abnormal emotions and behaviours due to altered hormone levels and neurotransmitter conduction in the body.

### Depression and anxiety

The diagnosis and treatment of infertility can cause emotional and psychological stress in patients, of which anxiety and depression are the most common mental disorders [35]. Aarts et al. [36] used the HADS and FertiQol to study 472 infertile women in the Netherlands. They found that the quality of life of the patients was low, and there was a negative correlation between anxiety and depression with quality of life. Kahyaoglu et al. [37] conducted a cross-sectional study of 85 infertile women with FertiQol and HADS and found that the quality of life of patients with anxiety and depression scores over 8 was significantly impaired. A study by Wishmann et al. [38] showed that the self-esteem of women with infertility decreased significantly, which led to a decrease in their sexual quality of life. Their overall quality of life was also significantly affected. In this study, the anxiety score was 54.84 ± 9.79 and the incidence was 69.34%, and the depression score was 58.22 ± 9.99, and the incidence was 83.21% (Table 2), indicating that the psychological state of RIF patients is not optimistic; furthermore, the findings of our study are worse than those of Lakatos et al. (anxiety rate 39.6%/depression rate 44.8%) [39]. The scores for the fertility QoL of RIF patients with anxiety and depression were significantly lower (57.13 ± 9.08 and 58.18 ± 8.68, respectively) (Table 2). We hypothesize that in China, the traditional ideology and societal gender orientation cause infertile women with RIF to experience pressure from family housework and working tasks, which increases their psychological and mental burden and seriously affects their fertility QoL. It has been reported in the literature [40] that during IVF-ET assisted pregnancy treatment, positive psychological support and counselling can effectively alleviate or eliminate psychological problems such as anxiety and depression, thus improving the health and QoL of patients with infertility and enhancing the treatment effects of infertility. It is suggested that when developing clinical technology, medical institutions should strengthen psychological counselling to provide RIF patients with appropriate opportunities to vent and alleviate their negative emotions. Similarly, health care professionals should strengthen health education, provide details of precautions and success rates during assisted pregnancy periods, and share successful cases of assisted pregnancy, thereby enhancing RIF patients’ confidence in assisted pregnancy and reducing their psychological distress. Furthermore, for medical centres with sufficient resources to effectively address psychological problems and improve the fertility QoL of RIF patients, psychological counselling clinics, hotlines, public WeChat, QQ groups, WeChat groups and other platforms can be established to help patients understand fertility and medical information.

### Family support

Martins et al. [41] investigated 252 infertile women under treatment and found that perceived family support directly or indirectly affected all aspects of stress related to fertility. The research of Yazdani et al. [42] showed that the quality of the relationship with their husband affects the marital satisfaction and marital quality of infertile women. This finding suggests that understanding and care from family members, the most intimate contact persons for patients, can help patients actively accept treatment, enhance their confidence to overcome the disease, improve their quality of life and promote rehabilitation. In this study, the total score of the social support scale was 60.92 ± 12.02, and the scores for family support and social support were most closely correlated with the scores of fertility QoL (Table 4) with a positive correlation (R = 0.745, R = 0.768). This finding indicates that the more family support and social support that patients experience, the better their fertility QoL. According to the results of multivariate regression analysis (Table 5), the standardized coefficient beta of family support was the highest, suggesting that family support had the greatest impact on fertility QoL. A study by Ching-Yu Cheng et al. noted that the relationship between infertile women and their spouses and family members can have a positive or negative impact on women's psychological stress and QoL during assisted pregnancy treatment [43]. Takaki and Hibino et al. also noted in their research in 2014 that a lack of family support can create stressful situations and increase psychological pressure on infertile women [44]. Sufficient family support can enable patients to obtain more care and emotional support and can improve their QoL and their ability to deal with psychological stress. Therefore, for infertile women undergoing assisted reproductive therapies, health care professionals should (1) comprehensively evaluate family function, (2) help to establish a better family support system, (3) encourage family members to participate actively in various health education activities, (4) create a good family atmosphere, and (5) give patients more support and care to improve the patient’s fertility QoL. It is also recommended that reproductive clinics can distribute missionary handbooks or play educational videos about assisted pregnancy and encourage couples to watch them together and discuss their feelings to reach mutual understanding and joint efforts in infertility treatment and the enhancement of family support, thereby improving their QoL and pregnancy rate.

**Table 4:** Correlation Analysis of Social Support, Anxiety, Depression and Fertility QoL in RIF Patients

Variables	Total Score of fertility QoL	Emotional response	Relationship between body and mind	Marital relations	Social relations	Therapeutic environment	Treatment tolerability
PSSS-Social support	0.768**	0.623**	0.601**	0.573**	0.782**	0.362**	0.334**
Family support	0.745**	0.537**	0.652**	0.528**	0.732**	0.434**	0.306**
Friend Support	0.649**	0.608**	0.528**	0.454**	0.678**	0.187*	0.254**
Other support	0.623**	0.496**	0.381**	0.535**	0.650**	0.326**	0.326**
SAS-Anxiety	− 0.503**	− 0.430**	− 0.358**	− 0.436**	− 0.488**	− 0.203**	− 0.255**
SDS-Depression	− 0.548**	− 0.528**	− 0.400**	− 0.475**	− 0.572**	− 0.093**	− 0.235**

Note: 1, **indicates P < 0.01, *indicates P < 0.05

2, 0.8–1.0 very strong correlation, 0.6–0.8 strong correlation, 0.4–0.6 moderate correlation, 0.2–0.4 weak correlation, 0.0–0.2 very weak correlation

**Table 5:** Multivariate Linear Regression Analysis of Influencing Factors of Fertility QoL of RIF Patients

Total Score of fertility QoL	Partial regression coefficient B	Standardized coefficient Beta	t-value	p value	Tolerance	VIF
(Constant)	55.267		9.776	0.000		
Residence = City	− 3.136	− 0.135	− 2.908	0.004	0.815	1.227
Self-Rating of financial difficulty = relative difficulty	− 4.473	− 0.193	− 3.327	0.001	0.518	1.929
Self-rated financial difficulties = great difficulty	− 10.655	− 0.377	− 5.829	0.000	0.420	2.381
Attribution of infertility = male factor	− 2.994	− 0.091	− 2.052	0.042	0.882	1.133
BMI index classification = < 18.5	− 8.011	− 0.142	− 3.027	0.003	0.798	1.253
BMI index classification = ≥ 24	2.822	0.091	2.066	0.041	0.898	1.113
Total score of family support	0.907	0.392	6.845	0.000	0.535	1.869
Standard score of depression	− 0.279	− 0.240	− 4.611	0.000	0.647	1.546
Total score of other support	0.438	0.152	2.724	0.007	0.564	1.774

Note: R^2^ = 0.777, adjusted R^2^ = 0.762, F = 49.253, P = 0.000. The regression model is established

## Limitations

In this study, we mainly focused on the external influencing factors of the fertility QoL of infertile women with RIF. However, some internal positive factors, such as resilience, may also affect the fertility QoL of RIF patients. This area of research and clinical implications deserves to be further explored. What’s more, further studies need to be conducted to examine whether the results of the present study are suitable to the different cultural context.

## Conclusion

This study found that the fertility QoL of infertile women with RIF was closely related to residence, male infertility, financial difficulties, BMI, depression, and family social support. As medical practitioners, we should understand patients’ positions and demands to achieve empathy. Moreover, it is necessary to create a good medical environment, respect the privacy of patients, and encourage patients to distract themselves to relieve their feelings. Finally, it is necessary to help patients establish a sound family and social support system. Through diverse types of health education, we can deepen patients’ and their family members’ understanding of infertility and encourage them to actively cooperate during treatment, thereby improving the therapeutic effects and the fertility QoL of patients.

## Data Availability

The datasets used and/or analysed during the current study are available from the corresponding author on reasonable request.

## Abbreviations

RIF: Repeated implantation failure
FertiQoL: Fertility quality of life
QoL: Quality of life
SD: Standard deviation
PSSS: Perceived Social Support Scale
SAS: Self-rating Anxiety Scale
SDS: Self-rating Depression Scale
COS: Controlled ovarian stimulation
IVF-ET: In vitro fertilization-embryo transfer
IVF: In vitro fertilization
ICSI: Intracytoplasmic sperm injection
BMI: Body mass index
